# Sibship Composition and BMI Z-Score Among Saudi Preschoolers: A Cross-Sectional Study

**DOI:** 10.7759/cureus.56485

**Published:** 2024-03-19

**Authors:** Rana H Mosli

**Affiliations:** 1 Department of Clinical Nutrition, Faculty of Applied Medical Sciences, King Abdulaziz University, Jeddah, SAU

**Keywords:** middle eastern, saudi, body mass index z-score, preschoolers, siblings

## Abstract

Background and objective

The association between sibship composition and child body mass index (BMI) has not been investigated in any Arab/Middle Eastern populations. In light of this, this study aimed to examine the association of the number of siblings, number of older siblings, and number of younger siblings with child BMI z-score (BMIz) among preschoolers in the Kingdom of Saudi Arabia (KSA).

Methods

A total of 209 mothers and their children were recruited from preschools in Jeddah, KSA. Mothers reported their responses to the study questionnaire via telephone. Child anthropometry was objectively measured; BMIz was calculated based on age- and sex-specific World Health Organization (WHO) growth standards and reference data. We used hierarchical multiple linear regression to examine the association between sibship composition variables and child BMIz, independent of the effect of potential confounders, and to evaluate changes in model fit.

Results

The number of siblings was negatively associated with child BMIz [b = -0.18, 95% confidence interval (CI) = -0.35, -0.06, p<0.01, adjusted R^2 ^= 0.16]. There was a negative association between the number of older siblings and child BMIz (b = -0.23, 95% CI = -0.38, -0.11, p<0.01, adjusted R^2 ^= 0.21) as well as between the number of older sisters and child BMIz (b = -0.18, 95% CI = -0.52, -0.09, p<0.01, adjusted R^2 ^= 0.19). However, there were no significant associations between the number of older brothers or the number of younger siblings and child BMIz.

Conclusions

Based on our findings, sibship composition was found to be associated with BMIz among a sample of preschoolers in KSA. More research is needed to further establish this association and to understand the underlying mechanism of the association of the greater number of older siblings and older sisters with lower BMIz.

## Introduction

Obesity remains a concern among various age groups in many countries worldwide [1]. A recent study investigating weight status among Saudi school-age children reported that 38% of children were overweight or obese [2]. Given the concurrent and long-term consequences of excess adiposity [3], researchers have been evaluating various contributing factors that may then be targeted as part of prevention and intervention efforts. Moreover, combating obesity among the youth requires efforts integrating both the school system and the household [4,5]. Within the household, several obesity risk factors have been identified, including diet quality and nutritional quality of meals, screen time, and physical activity levels [6,7]. In addition, interaction among family members around food has also received ample attention as a factor associated with child eating behaviors and weight status [8]. Evaluation of these relationships has highlighted the important role that family members may play in shaping child outcomes. Family structure and functioning have been found to predict child weight status and may be important factors to consider when planning obesity treatment and intervention programs [4].

Evidence that sibship composition is associated with child body mass index (BMI) has also been reported from various countries worldwide [9-14]. However, findings related to this have been inconsistent owing to some methodological distinctions and possible cultural variations. Results from studies in the United States (US) [9-12], Australia [13], Japan [14,15], China [16], and Denmark [17], as well as a study involving eight European countries [18], have suggested an inverse association between the number of siblings and the likelihood of overweight or obesity among children, with singletons or only children having the greatest odds of being obese. Other research has found a positive [19,20] or no association [21,22] between the number of siblings and BMI. These studies include those conducted in Eastern European countries (i.e., Poland) [19] and South American countries (i.e., Brazil) [21], and those involving participants of older age groups [20,22].

Moreover, several studies have reported that having fewer siblings may be associated with poorer dietary habits and a lower physical activity level [23-25]. Others have suggested a greater likelihood of maladaptive maternal feeding behaviors with having fewer siblings [24,26,27]. However, it is still unclear whether the effect of the number of siblings differs in terms of whether these siblings are younger or older than the focal child. Evidence suggests that having a larger number of younger siblings (rather than older siblings) is associated with lower child BMI [6,9,10,14]. However, some reports have shown that having more younger siblings may be associated with higher BMI [19], while others have found that a larger number of older siblings correlates with lower child BMI [15].

Family functioning, including sibling relationships, has been found to differ among cultures [28,29]. Thus, it is plausible that the effect of sibship-related circumstances on child BMI may also vary among cultures. Like several other Middle Eastern countries, the Kingdom of Saudi Arabia (KSA) is undergoing rapid economic and societal transitions [30]. Obesity rates among children in KSA are on the rise, particularly among higher-income youths [31]. However, the association between the home environment, particularly family characteristics, and child BMI has not been extensively studied. We were unable to find any studies in the literature focusing on the relationship between sibship circumstances and child weight status in a Saudi or any other Arab sample. A recent study only reported that having a “small family (two to five members) in the house” was linked to a higher prevalence of overweight or obesity among Saudi school-age children [2]. Establishing this relationship can help design targeted obesity treatment programs for families with Arab or Middle Eastern backgrounds.

Thus, this study aimed to examine the association of sibship composition (i.e., number of siblings, number of older siblings, and number of younger siblings) with child BMI z-score (BMIz) among preschool children in KSA while adjusting for various covariates.

## Materials and methods

Sample size and procedures

The participants were randomly recruited from the city of Jeddah, KSA, by using stratified sampling based on geographic location and type of preschool (public vs. private). A total of 209 children from eight different preschools were included: two preschools from the Northern area, two from the Southern area, two from the Eastern area, and two from the Western area. Of the eight preschools, four were public (government-subsidized), and four were privately run. We placed a description of the study and consent forms in the backpacks of all students attending the preschools (n = 1,783 enrolled students). Mothers who returned signed consent forms were contacted via telephone by research assistants to complete the study questionnaire. Then, within a week following the completion of the questionnaire, the study team performed school visits and completed the collection of child anthropometric measurements. The following criteria had to be met by mother-child dyads to be enrolled in the study: the child was Saudi or a permanent resident of SA; aged three to six years; lived primarily with his/her mother and was considered “healthy” with no serious medical issues or history of food allergies; and the mother understood and spoke the Arabic language fluently. The ethical approval to conduct this study was obtained from the Unit of Biomedical Ethics at King Abdulaziz University (reference no: HA-02-J-008).

Measures

Primary Predictors: Sibship Composition Variables

Mothers were asked to answer questions about sibship composition, including the total number of siblings the index child has, as well as the total number of older siblings, the total number of older sisters, the total number of older brothers, the total number of younger siblings, the total number of younger sisters, and the total number of younger brothers.

Primary Outcome: Child BMIz

During preschool visits, children’s weights and heights were measured by using standardized procedures; The child was asked to remove shoes, any heavy clothing (e.g., jacket), and hair ornaments. A calibrated digital scale was used to measure weight, and each child was made to stand on the center of the scale. The weight was recorded to the nearest decimal fraction. A stadiometer was used to measure height. Each child was made to stand with their back to the stadiometer and with feet together and flat. BMI was calculated for the participating children by dividing their weight (kg) by height (m^2^). BMIz for each child was estimated using the age- and sex-specific World Health Organization (WHO) growth standards for children <5 years old and the WHO growth reference data for children aged five to six years [32,33].

Covariates

Data on children's demographic characteristics, such as sex, birthdate, and nationality, were collected from mothers through the study questionnaire. Mothers also reported the child’s birth weight in kg, as well as information regarding their own birthdates, educational level, employment status, and the monthly income of the family. The child’s and mother’s ages were later calculated based on birthdates and dates of interviews. Questions assessing the mother’s current weight and height were also included. Maternal BMI was calculated by dividing weight (kg) by height (m^2^) [10].

Statistical analysis

Data were analyzed using IBM SPSS Statistics 21.0 (IBM Corp., Armonk, NY). Characteristics of the sample including demographic and child characteristics were examined using descriptive statistics; means (M) and standard deviations (SD) were calculated for continuous variables and counts and percentages were calculated for categorical variables.

The associations of demographic and child variables with child BMIz and number of siblings were assessed, including sex (male vs. female), nationality (Saudi vs. non-Saudi), maternal education (<college education vs. ≥college education), maternal employment status (employed vs. unemployed), and total monthly income ( ≤10,000 SR vs. >10,000 SR) (10,000 SR is equivalent to 2,666 USD). Differences in mean child BMIz and mean number of siblings between groups were examined using independent samples t-test. Pearson correlation was used to examine the association of the continuous variables - child age, child birthweight, maternal age, and maternal BMI - with child BMIz and number of siblings. Demographic and child-related variables that were found to be associated with both child BMIz and number of siblings (using a more conservative cutoff of p<0.10), including total monthly income, child nationality, child age, child birth weight, and maternal BMI, were considered to be potential confounders. Each of these potential confounders was later used as covariates in the regression models.

We used hierarchical multiple linear regression to examine the association between sibship composition variables and child BMIz, independent of the effect of covariates, and to evaluate the change in model fit (i.e., adjusted R^2^) [34]. Furthermore, to examine the effect moderation by child sex, we tested the interaction term for child sex and the number of siblings in each of the fully adjusted models. The significance level was set at p<0.05.

## Results

Sample characteristics

The mean age of the children in the study was 4.79 years (SD = 0.79), and the mean child BMIz was 0.16 (SD = 1.33). Approximately half of the children in the sample (n = 108, 51.7%) were male, and the majority (n = 145, 69.4%) of them were Saudi. Among the mothers participating in the study, more than half (n = 143, 68.4%) reported receiving at least a college-level education, and approximately half (n = 100, 48%) categorized themselves as “housewives”. The total family income was 10,000 SR or less per month for 45.5% (n = 95) of the sample (Table 1).

**Table 1:** Demographics and child characteristics (n = 209) BMI: body mass index; SD: standard deviation

Variable	Value
Child sex, n (%)	
Male	108 (51.7)
Female	101 (48.3)
Age, mean (SD)	4.79 (0.79)
Child nationality, n (%)	
Saudi	145 (69.4)
Non-Saudi	64 (30.6)
Child birth weight, kg, mean (SD)	2.93 (0.65)
Maternal age, mean (SD)	33.1 (4.98)
Maternal education, n (%)	
< College education	66 (31.6)
≥ College education	143 (68.4)
Maternal employment, n (%)	
Employed	102 (48.8)
Unemployed	107 (51.2)
Total monthly income, n (%)	
≤ 10,000 SR	95 (45.5)
> 10,000 SR	114 (54.5)
Maternal BMI, mean (SD)	26.6 (5.34)

Association of child and maternal characteristics with child BMIz

As shown in Table 2, non-Saudi children had higher BMIz compared to Saudi children (M = 0.62, SD = 1.27 vs. M = -0.04, SD = 1.32, p<0.01). Furthermore, children with a family monthly income >10,000 SR per month had higher BMIz compared to children with a family income of 10,000 SR per month or less (M = 0.45, SD = 1.29 vs. M = -0.17, SD = 1.31, p<0.01). Although not reaching statistical significance, there was a negative correlation between child age and child BMIz (r = -0.12, p<0.10). However, there was a positive correlation between child birthweight and child BMIz (r = 0.20, p<0.05) as well as between maternal BMI and child BMIz (r = 0.14, p<0.05).

**Table 2:** Association of demographic and child characteristics with child BMIz and number of siblings (n = 209) *P-value <0.05. **P-value <0.01. tP value <0.1 Correlation of continuous variables with child BMIz and number of siblings examined using Pearson correlation. Variables that had an association with both child BMIz and number of siblings (p<0.10) (i.e., child nationality, total monthly income, child age, child birth weight, and maternal BMI) were considered as potential confounders BMIz: body mass index z-score; SD: standard deviation

Analysis of the association of demographic and child characteristics with child BMIz and number of siblings		
Differences in mean child BMIz and number of siblings		
	Child BMIz, mean (SD)	Number of siblings, mean (SD)
Child sex		
Male	0.26 (1.45)	1.66 (1.14)
Female	0.06 (1.20)	1.90 (1.29)
Child nationality		
Saudi	-0.04 (1.32)^**^	1.89 (1.21)^*^
Non-Saudi	0.63 (1.27)	1.52 (1.20)
Maternal education		
< College education	0.19 (1.35)	1.85 (1.14)
≥ College education	1.56 (1.34)	1.74 (1.26)
Maternal employment		
Employed	0.25 (1.28)	1.71 (1.24)
Unemployed	0.08 (1.39)	1.84 (1.21)
Total monthly income		
≤ 10,000 SR	-0.17 (1.31)^**^	1.89 (1.38)^*^
> 10,000 SR	0.45 (1.29)	1.68 (1.06)
Correlations with child BMIz and number of siblings		
	Correlation with child BMIz	Correlation with the number of siblings
Child age	-0.12^t^	0.21^**^
Child birth weight	0.20^**^	-0.18^**^
Maternal age	-0.09	0.35^**^
Maternal BMI	0.14^*^	0.19^**^

Associations of child and maternal characteristics with the number of siblings

All variables that were found to be significantly associated with child BMIz were also significantly associated with the number of siblings; Saudi children had more siblings compared to non-Saudi children (M = 1.89, SD = 1.21 vs. M = 1.52, SD = 1.20, p<0.05). Additionally, children with a family monthly income >10,000 SR per month had fewer siblings compared to children with a family income of 10,000 SR per month or less (M = 1.68, SD = 1.06 vs. M = 1.89, SD = 1.06, p<0.05). The number of siblings was positively correlated with child age (r = 0.21, p<0.01) and maternal BMI (r = 0.19, p<0.01), but negatively correlated with child birthweight (r = -0.18, p<0.01) (Table 2).

Association between the number of siblings and child BMIz

Controlling for potential confounders (i.e., child, age, child nationality, child birth weight, total monthly income, and maternal BMI), the number of siblings was negatively associated with child BMIz [b = -0.18, 95% confidence interval (CI) = -0.35, -0.06, p<0.01], and this model explained 16% of the variance in child BMIz. A stronger association was observed when the “number of older siblings” was included as the primary predictor (b = -0.23, 95% CI = -0.38, -0.11, p<0.01), with an increase of 5% in the adjusted R^2^, indicating a better model fit. We found a negative association between the number of older sisters and child BMIz (b = -0.18, 95% CI = -0.52, -0.09, p<0.01). However, the association between the number of older brothers and child BMIz did not reach statistical significance (Table 3). We did not detect a significant association between any of the other sibling-related variables and child BMIz. Moreover, there was no significant association between child sex and any of the primary predictors in the fully adjusted models (p>0.10).

**Table 3:** Association between the number of siblings and child BMIz *Child nationality (Saudi vs. non-Saudi), total monthly income (>10,000 SR vs. ≤10,000), child age, child birth weight, and maternal BMI were forced into the models before adding sibling variables. Standardized b coefficients for the control variables were -0.12 (p = 0.09), -0.23 (p = 0.001), 0.20 (p = 0.005), 0.14 (p = 0.04), and 0.23 (p = 0.001), respectively BMIz: body mass index z-score

Primary predictor	Change in R^2*^	Standardized b coefficient (95% CI)	P-value
Number of siblings	0.03^*^	-0.18 (-0.35, -0.06)	0.007
Number of older siblings	0.05^*^	-0.23 (-0.38, -0.11)	0.001
Number of older sisters	0.03^*^	-0.18 (-0.52, -0.09)	0.005
Number of older brothers	0.01	-0.10 (-0.41, 0.05)	0.12
Number of younger siblings	0.004	0.06 (-0.13, 0.36)	0.35
Number of younger sisters	0	0.01 (-0.27, 0.29)	0.93
Number of younger brothers	0.006	0.08 (-0.14, 0.58)	0.22

## Discussion

This study involving 209 mother-child dyads is the first to report evidence of an association between sibship circumstances and child BMIz among an Arab/Middle Eastern sample. Controlling for potential confounders (i.e., child age, child nationality, child birth weight, total monthly income, and maternal BMI), we found that a greater number of siblings was associated with lower child BMIz. Further analyses revealed that this association was specific to older (rather than younger) siblings. Namely, we found that an increase in the number of older sisters (rather than older brothers) was associated with lower child BMIz. Our analysis suggests that the association between sibship circumstances and child BMIz is not affected by the index child’s sex.

Only one previous study, involving a Japanese cohort, has reported that having a greater number of older siblings or sisters was linked to lower child BMI; however, this study only detected this association among male children [15]. Although several other studies from various countries have found that having more siblings was inversely associated with child BMI [9,10,14,17,18], none has reported the association to be specific to older siblings or older sisters. Conversely, studies in the US have reported that having a greater number of younger siblings (rather than older) was associated with lower obesity risk [9-11]. Furthermore, a study including a low-income US sample has found that having at least one brother was associated with lower odds of child obesity, but no association was found between having at least one sister and child obesity [10]. Our findings are in line with a previous study including school-age children in KSA, which reported that having a “small family (two to five members) in the house” was linked to a higher prevalence of overweight or obesity among children [2].

Having siblings may significantly impact children’s behavior and development, either directly through interactions between siblings, or indirectly through modifications in parental interactions with the child [26,35-38]. Several possible mechanisms may explain the relationship between sibship composition and child weight status. First, a greater number of siblings may be associated with the child being more physically active, and thus having a higher energy expenditure throughout the day [24]. Second, mothers may employ more structured mealtimes and use less restrictive feeding behaviors when a greater number of siblings are present [26]. Finally, siblings may act as role models and caregivers, which may positively impact the child’s eating behavior [39].

Our study’s findings suggest that the association between sibship composition, particularly when taking into account siblings’ age and sex, may not be constant across different communities. Therefore, each of the underlying mechanisms mentioned above needs to be explored distinctly in different cultures. Indeed, mealtimes are usually regarded as a “vehicle for culture” - an occasion during which family members engage in the activity of eating and feeding, as well as shape and reinforce social order [40]. Therefore, the effect of sibship composition on child BMI through interactions among siblings and parents during mealtimes may vary across cultures. Furthermore, family dynamics and house rules around the use of electronics and physical activity may impact the degree of siblings’ engagement and modeling of these behaviors [41]. Further research is needed to understand the underlying mechanism of the association between a greater number of older siblings and older sisters with lower child BMI in Arab/Middle Eastern communities.

Establishing the association between sibship composition and child BMI and understanding the underlying mechanisms can help in designing targeted family-based programs for children from Arab/Middle Eastern backgrounds. Findings can aid practitioners and researchers in adopting a culturally sensitive approach that considers unique family dynamics and optimizes the integration of various family members [42]. Such efforts can help reduce obesity rates and promote a healthy weight status among children.

Strengths and limitations

The strengths of our study include the fact that weight and height data were collected from children through objective measurements using standardized procedures. Furthermore, mother-child dyads were recruited through random selection from preschools with varying levels of income. The study has certain limitations as well. Our sample was relatively small. This might have influenced our statistical power to identify significant associations. In addition, our results may not be generalizable to families where mothers have lower educational levels, given the high proportion of mothers with a college education in our sample.

## Conclusions

Sibship-related factors may be significantly associated with child BMIz in Arab/Middle Eastern families. Having more older siblings and older sisters may be linked to lower weight status. Future studies with larger sample sizes that include mothers with various educational levels are needed. Longitudinal designs may be useful in evaluating the effect of sibship composition on child weight status over time and understanding the underlying mechanisms of association. Qualitative data and assessment of family functioning and interactions through direct observation during mealtimes as well as active play may also aid in establishing the pathway between sibship composition and child BMI. Findings from such research can help implement family-focused programs to promote healthy weight status among children.

## Appendices

**Table 4:** Study questionnaire

Question	Response
Questions regarding the child who is participating in the study:	
1. What is the child’s date of birth? (DD/MM/YYYY)	
2. What was your child’s birth weight?	___ kg
Questions regarding family structure:	
3. How many family members live in your household?	
4. How many siblings does your child have? (if none enter 0 and go to Q9)	
5. How many older sisters does your child have?	
6. How many older brothers does your child have?	
7. How many younger sisters does your child have?	
8. How many younger brothers does your child have?	
Questions regarding the mother’s (your) characteristics:	
9. What is your date of birth? (DD/MM/YYYY)	
10. What is your current weight?	___ Kg
11. What is your current height?	___ cm
12. What is the highest educational level you have received?	1. Illiterate
	2. Literate
	3. Primary school
	4. Middle school
	5. High school
	6. College
	7. Postgraduate degree
	8. Other:
13. Please specify your employment status:	1. Housewife
	2. Employed
	3. Student
	4. Other:
14. If you are an employee/student, please specify the number of hours you work per day	1. Less than 4 hours
	2. 4-8 hours
	3. More than 8 hours
	4. Does not apply
15. Please specify your marital status	1. Married
	2. Divorced
	3. Separated
	4. Widowed
	5. Other:
Questions regarding demographic characteristics	
		16. Does your child hold Saudi nationality?	1. Yes
2. No		
17. Do you hold Saudi nationality?	1. Yes
	2. No
18. What is your family’s total monthly income?	1. Less than 5000 SR
	2. 5000 to 10000 SR
	3. More than 10000 SR
